# High-Throughput Screening for Novel Inhibitors of *Neisseria gonorrhoeae* Penicillin-Binding Protein 2

**DOI:** 10.1371/journal.pone.0044918

**Published:** 2012-09-25

**Authors:** Alena Fedarovich, Kevin A. Djordjevic, Shauna M. Swanson, Yuri K. Peterson, Robert A. Nicholas, Christopher Davies

**Affiliations:** 1 Department of Biochemistry & Molecular Biology, Medical University of South Carolina, Charleston, South Carolina, United States of America; 2 Department of Microbiology and Immunology, University of North Carolina at Chapel Hill, Chapel Hill, North Carolina, United States of America; 3 Department of Pharmaceutical and Biomedical Sciences, Medical University of South Carolina, Charleston, South Carolina, United States of America; 4 Department of Pharmacology, University of North Carolina at Chapel Hill, Chapel Hill, North Carolina, United States of America; La Trobe University, Australia

## Abstract

The increasing prevalence of *N. gonorrhoeae* strains exhibiting decreased susceptibility to third-generation cephalosporins and the recent isolation of two distinct strains with high-level resistance to cefixime or ceftriaxone heralds the possible demise of β-lactam antibiotics as effective treatments for gonorrhea. To identify new compounds that inhibit penicillin-binding proteins (PBPs), which are proven targets for β-lactam antibiotics, we developed a high-throughput assay that uses fluorescence polarization (FP) to distinguish the fluorescent penicillin, Bocillin-FL, in free or PBP-bound form. This assay was used to screen a 50,000 compound library for potential inhibitors of *N. gonorrhoeae* PBP 2, and 32 compounds were identified that exhibited >50% inhibition of Bocillin-FL binding to PBP 2. These included a cephalosporin that provided validation of the assay. After elimination of compounds that failed to exhibit concentration-dependent inhibition, the antimicrobial activity of the remaining 24 was tested. Of these, 7 showed antimicrobial activity against susceptible and penicillin- or cephalosporin-resistant strains of *N. gonorrhoeae*. In molecular docking simulations using the crystal structure of PBP 2, two of these inhibitors docked into the active site of the enzyme and each mediate interactions with the active site serine nucleophile. This study demonstrates the validity of a FP-based assay to find novel inhibitors of PBPs and paves the way for more comprehensive high-throughput screening against highly resistant strains of *N. gonorrhoeae*. It also provides a set of lead compounds for optimization of anti-gonococcal agents.

## Introduction

Historically, β-lactams have been highly successful for the treatment of bacterial infections, but the emergence of resistance to these and other antibiotics has markedly limited the treatment options in a number of pathogens. One notable example is *Neisseria gonorrhoeae*, an obligate human pathogen and cause of the sexually transmitted disease, gonorrhea. For over 40 years, gonorrhea was treated with a single dose of penicillin, but the increasing prevalence of strains exhibiting resistance to penicillin eventually led to its withdrawal in 1986 by the Centers for Disease Control and Prevention (CDC) as a recommended antibiotic for gonococcal infections. With tetracycline and fluoroquinolones [1] being withdrawn for similar reasons, only the extended-spectrum cephalosporins, cefixime and ceftriaxone, remain as recommended treatments in the U.S. However, during the last decade, strains exhibiting decreased susceptibility to cefixime and ceftriaxone have emerged in Japan and Europe [2], [3], [4], [5], [6], [7], and the recent isolation of two distinct strains in Japan and France with high-level cefixime and ceftriaxone resistance (both with minimum inhibitory concentrations (MICs) ≥2 µg/ml)) [8], [9] signals the potential demise of these antibiotics as effective treatments for gonorrhea [10].

The lethal targets for β-lactams are penicillin-binding proteins (PBPs) [11],[12], which are transpeptidases that catalyze the formation of peptide cross-links between adjacent glycan strands during the final stages of peptidoglycan synthesis in bacteria. Peptidoglycan envelops the bacterial cell and is essential for cell growth, division and maintenance of cell shape [13]. PBPs share a common penicillin-binding/transpeptidase domain with an active site that contains three conserved motifs: SxxK, SxN and KTG (where × is a variable residue) [14]. The SxxK motif contains the serine nucleophile that attacks the carbonyl carbon of the penultimate d-Ala of the peptide substrate or amide carbonyl of the β-lactam ring. There are three classes of PBP: Class A PBPs catalyze both glycosyl transferase and transpeptidase activities, Class B catalyze only transpeptidase activity and Class C PBPs are carboxypeptidases and/or endopeptidases. In most bacteria, Class A and B PBPs are essential enzymes, whereas Class C PBPs can often be deleted genetically without significant impact on cell growth or morphology [15]. The genome of *N. gonorrhoeae* encodes 4 PBPs. PBPs 3 and 4 are Class C PBPs and are non-essential for cell viability [16]. PBP 1 (Class A) and PBP 2 (Class B) are both essential, but given that PBP 2 is inhibited at a 10-fold lower concentration of penicillin than PBP 1, it is the primary clinical target in penicillin-susceptible strains [17], [18].

*N. gonorrhoeae* develops chromosomally mediated resistance to β-lactams through alteration of the PBP targets, increased expression of the MtrC-MtrD-MtrE efflux pump and mutation of the porin PorB1b that restricts entry into the periplasm [19], [20]. The primary step in this process is the acquisition of mutated forms of PBP 2 that exhibit lowered reactivity with β-lactams and compromise the effectiveness of these agents [21], [22], [23], [24], [25], [26]. PBP 2 is essential for the growth of *N. gonorrhoeae* and is a validated target for β-lactam antibiotics directed against this organism [18], but its value as a clinical target has been diminished by mutations associated with resistance. In order to develop new treatment options for penicillin- and cephalosporin-resistant strains of *N. gonorrhoeae*, new inhibitors of PBP 2 are needed. In this study, we report the development of a high-throughput screening (HTS) assay for PBPs that uses fluorescence polarization to detect binding of the fluorescent β-lactam, Bocillin-FL [27]. We used this assay to screen a 50,000 chemical library and identified a number of compounds that inhibited PBP 2 activity in the micromolar range. Of these, seven demonstrated antimicrobial activity against *N. gonorrhoeae*, including strains exhibiting resistance to penicillin or cefixime.

## Materials and Methods

### Materials

A soluble construct of wild-type PBP 2 from the penicillin-susceptible strain of *N. gonorrhoeae* FA19 was expressed and purified as described previously [26]. Bocillin FL™ was obtained from Invitrogen Inc. (Carlsbad, CA). Penicillin G and γ-Globulins from bovine blood (BGG) were purchased from Sigma (St. Louis, MO). Prior to use, all reagents were diluted in an assay buffer comprising 50 mM potassium phosphate, pH 8, and 0.1 mg/ml BGG. The DIVERSet library of 50,080 small lead compounds from ChemBridge Corporation (San Diego, CA) was provided by the MUSC Drug Discovery Core (DDC). Three laboratory strains of *N. gonorrhoeae*–FA19 [28], penicillin-resistant FA6140 [29], and cephalosporin-intermediate resistant Ceph^I^ strain 35/02 [30]–were from the laboratory collection of R. Nicholas (UNC-Chapel Hill).

### Fluorescence Polarization (FP) Measurements

Measurements of fluorescence and FP were performed on a Spectramax M5 microplate reader (Molecular Devices, Sunnyvale, CA) with excitation and emission wavelengths of 485 nm and 520 nm, respectively, using a 515 nm filter to block residual excitation light and to minimize background interference. Black, shallow 384-well micro plates (ProxiPlate ™ –384 F Plus, PerkinElmer) were used to record data. To minimize the polarization effects from any fluorophore bound to the surface of the well, both excitation and emission data were recorded from the top of the well. Reading time was 100 ms per well. Fluorescence polarization was quantified in millipolarization units (mP) according to the equation, mP = 1000×[(I_v_ − G.I_h_)/(I_v_ + G.I_h_)], where I_v_ and I_h_ are parallel and perpendicular emission intensity measurements, respectively, corrected for background (buffer), and G is the instrument-dependent correction coefficient, defined as a ratio of sensitivities of the detection system for vertically and horizontally polarized light. Using the equation G  =  I_v_/I_h_ and SoftMax® Pro Data Acquisition & Analysis Software (Molecular Devices, Sunnyvale, CA), the G-factor was determined with dilution series of Bocillin-FL-only (free tracer). The assay window (ΔmP) was defined as the difference between protein-tracer sample and free-tracer, *i.e.* ΔmP  =  mP_s_ – mP_free_, and is a measure of the maximum specific binding.

### FP Assay Optimization

To calculate the G-factor, FP was measured in 10 µl reaction volumes for free Bocillin-FL at concentrations of 0.2, 0.5, 1, 2, 3, and 4 µM, where the FP signal of the fluorescent tracer was low and stable. The optimal tracer-to-protein ratio was determined in the binding experiments with increasing concentrations of PBP 2 (0.02–4 µM). FP was recorded after shaking the plate for 2 min followed by 30 min incubation, at which point the reaction reached its steady state (data not shown). Each experiment was performed in quadruplicate at room temperature.

To evaluate the performance of the assay, steady-state concentration-response experiments were carried out using penicillin G in a competition assay with Bocillin-FL. Penicillin G (0.05–1000 µM) was mixed with 1 µM PBP 2 and 1 µM Bocillin-FL, followed by a 1 hr incubation. The positive (Pc) and negative (Nc) controls were defined as the FP of the Bocillin-FL - protein and of the free tracer, respectively, in the absence of penicillin G. The FP of the Bocillin-FL - protein at 100 µM penicillin G was defined as a displaced tracer control (Dc). Since DMSO was used as a solvent in the compound library, the effect of 10% DMSO on the FP-binding assay was also determined. Data points were normalized to the maximum specific binding, which defines complete saturation of PBP 2 by Bocillin-FL in the absence of penicillin G, and IC_50_ values were determined using non-linear regression analysis using GraphPad Prism version 4.00 for Windows (GraphPad Software, Inc, San Diego, CA).

Assay performance was assessed using the following parameters: the signal-to-noise ratio S/N  =  (µ_pc_-µ_nc_)/SD_nc_, Z′ and Z factors. The latter were calculated as Z′ = 1− (3SD_pc_ +3SD_nc_)/(µ_pc_-µ_nc_) and Z = 1− (3SD_pc_ +3SD_dc_)/(µ_pc_-µ_c_), where SD_pc_, SD_nc_, SD_dc_ are standard deviations and µ_pc_, µ_nc_, µ_dc_ are means of recorded polarization values of Pc, Nc, and Dc, respectively [31].

### High-throughput Assay and Screening for the Inhibitors

HTS screening against the ChemBridge DIVERSet library was carried out under the following conditions: 1 µl of each compound (10% DMSO final) in duplicate was pre-incubated with 9 µl of PBP 2 for 1 h at room temperature, followed by additional 30 min incubation with 2 µl Bocillin-FL (0.87 µM PBP2∶ 1 µM Bocillin-FL final). In addition to samples with the compounds, each plate also contained 2 background wells (12 µl buffer only), and at least 4 wells each for Pc, Nc, and Dc reactions. All samples used for background measurements or controls included 10% DMSO. Initial screening was carried out with 10 mM cocktails of 10 compounds (giving a final concentration of 100 µM for each compound). Compounds from cocktails that showed 80% or more reduction in ΔmP compared to maximum binding (ΔmP_max_  =  Pc – Nc) were then tested individually. The auto fluorescence of each compound was also tested individually.

### Concentration-response Experiments

Concentration-response experiments with the individual compounds that demonstrated ≥50% ΔmP reduction compared to maximum binding were conducted using FP-based and SDS-PAGE-based steady-state binding assays. For the FP experiments, 0.01–200 µM of each potential hit was incubated with PBP 2 (1 µM) for 1 h, followed by an additional 30 min incubation with 1 µM Bocillin-FL to detect the residual activity of the labeled penicillin binding. For the SDS-PAGE-based concentration-response experiments, PBP 2 (1 µM) in 50 mM sodium phosphate, 0.01% Triton X-100, pH 8 was incubated with 0.05–1000 µM of a compound for 1 h, followed by additional 15 min incubation with 10 µM Bocillin-FL. The reaction was stopped by mixing each sample with 5 X SDS-loading buffer, followed by boiling for 2 min. The samples were submitted to electrophoresis on 10% Mini-Protean TGX™ SDS-PAGE gels (Bio-Rad, Hercules, CA) to separate bound PBP 2 from free ligand. At least two independent reactions were performed in duplicate at each concentration of an inhibitor. Gels were visualized by UV using a Kodak EDAS 290 imaging system (Scientific Imaging Systems Eastman Kodak, New Haven, CT, USA) and the protein bands were stained with Coomassie to verify equal loading. Densitometry was performed with ImageJ 1.37v program (National Institutes of Health, USA, http://rsb.info.nih.gov/ij/). In both methods, data points were normalized to the maximum value of the fluorescence intensity, which defines complete saturation of PBP 2 by Bocillin-FL in the absence of compound, and IC_50_ values of the inhibitors were defined as the concentration of a compound at 50% of the residual activity of Bocillin-FL and calculated using GraphPad Prism (see above).

### Antimicrobial Susceptibility Testing

Three strains of *N. gonorrhoeae* were used in susceptibility tests: 1) FA19, a penicillin-and cephalosporin-susceptible strain; 2) FA6140, a penicillin-resistant but cephalosporin-susceptible strain; and 3) 35/02, a penicillin-resistant and cephalosporin intermediate-resistant strain. The disc diffusion zone method was applied for preliminary testing of “hits” identified in the screening process [32]. For this method, 100 µl of a cell suspension (∼1×10^7^ bacteria) was added to 3 ml of GCB top agar (GC Broth containing 0.75% agar) at 50^o^C, which was then added to GCB plates and allowed to solidify. Five µl of each compound (5 µg) was added separately to antibiotic susceptibility discs (BBL™, BD, Franklin Lakes, NJ), the discs were placed on top of plates, and the plates were incubated overnight in a 4% CO_2_ incubator at 37^o^C. Those compounds that produced a visible zone of inhibition were then tested for their MICs on agar plates as described [33]. Briefly, GCB agar plates containing increasing amounts of the tested compounds were spotted with 5×10^4^ cells. The lowest concentration of the test compound that prevented growth of <5 colonies was defined as the MIC. These experiments were repeated three times, with each experiment producing similar results.

### Molecular Modeling of PBP2 Inhibitors

Modeling, simulations and visualizations were performed using Molecular Operating Environment (MOE) Version 2011.10 (Chemical Computing Group Inc., Montreal, Canada). The crystal structure of wild-type *N. gonorrhoeae* PBP 2 was used as the starting model for docking simulations (PDB:3EQU; [26]). Following protonation of the main chain at pH 7.5 and addition of salt at a concentration of 0.2 M, the structure was energy minimized using the Amber99 forcefield and Born solvation model. Ligands corresponding to the 7 compounds that displayed antimicrobial activity against *N. gonorrhoeae* were prepared in MOE, and also protonated at pH 7.5 with a salt concentration of 0.2 M. The entire protein surface was used for the simulations, in which PBP 2 was held rigid and the ligand was flexed. Five hundred poses per ligand were derived using triangle-matching placement with London dG scoring. The top 250 poses were then refined using forcefield placement and affinity dG scoring, leading to a final database of 1750 poses for the seven compounds. All poses within the active site were then energy minimized using the Amber99 forcefield and Born solvation model, with both PBP 2 and the ligand allowed to flex.

## Results

### Development of the Fluorescence Polarization Assay

**Figure 1:**
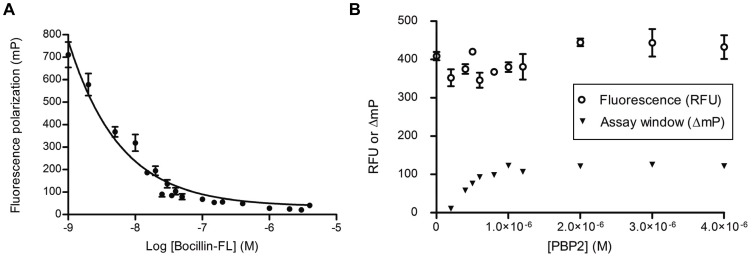
Optimization of the fluorescence polarization (FP) assay. **A**. Fluorescence polarization (mP) of free Bocillin-FL at various concentrations from 0.002 to 4 µM. **B**. Binding experiments with Bocillin-FL (1 µM) and PBP 2 at various protein concentrations from 0.2 to 4 µM. Fluorescence of Bocillin-FL – PBP2 was measured in relative fluorescence units (RFU) (open circles). Maximum specific binding (triangles), *i.e.* assay window (ΔmP), was determined by FP. Assay window is defined as the difference between FP of protein-tracer sample and free-tracer, *i.e.* ΔmP  =  mP_s_ – mP_free_. In all experiments, the data points represent the mean ± standard deviation of four replicate experiments at each concentration of Bocillin-FL or PBP 2.

Initial experiments were performed to elucidate the minimal amounts of Bocillin-FL and PBP 2 that give the largest range of specific binding. The mP recorded for free Bocillin-FL in the range of 0.2–4 µM was 40–65 mP (Fig. 1A). Based on the results of steady-state binding experiments using variable concentrations of PBP 2 and Bocillin-FL, the optimal assay window (ΔmP) was 119 mP at a protein-Bocillin-FL ratio of 1∶1 µM. At this ratio, the total fluorescence of Bocillin-FL was not significantly affected by protein binding (Fig. 1B). The average value of the G-factor coefficient used to correct mP measurements for the instrument bias was 1.0±0.01. This was determined within a 0.2–4 µM concentration range of free Bocillin-FL and used in subsequent FP experiments, including the HTS.

**Figure 2:**
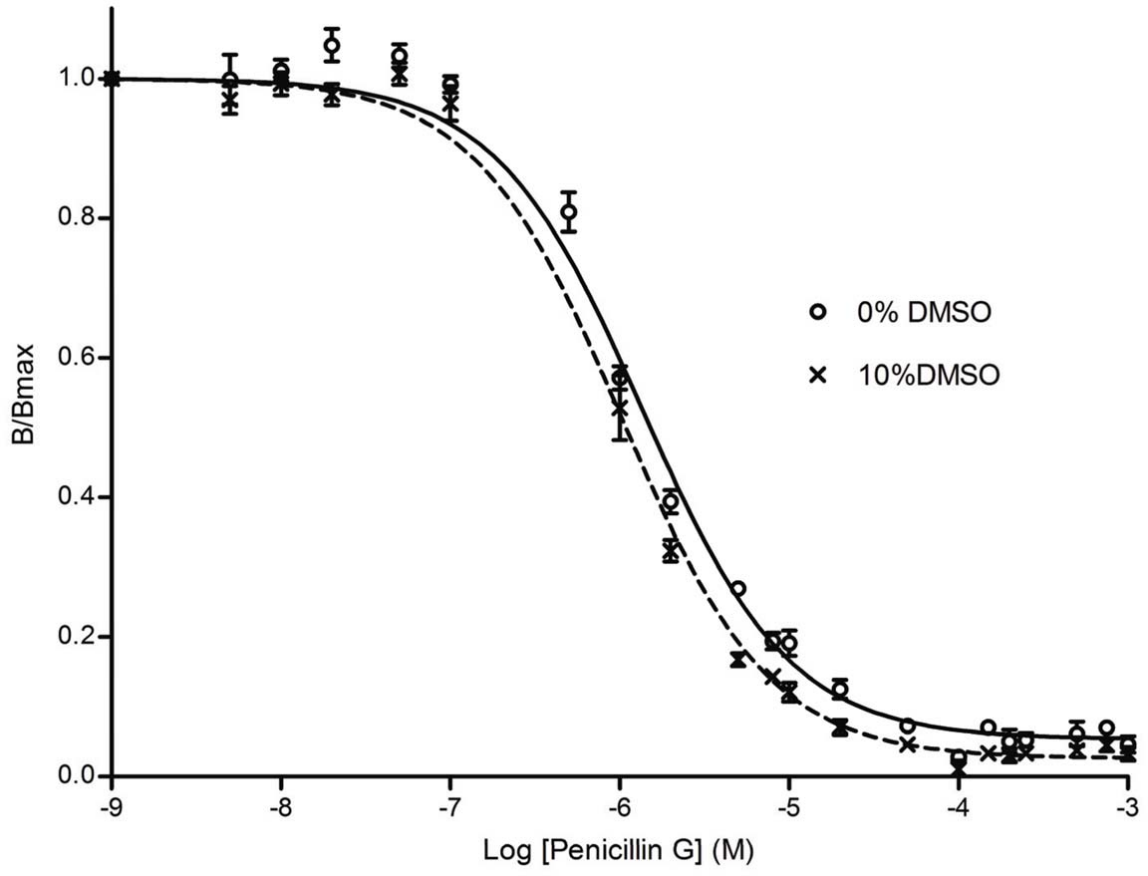
Inhibition of FP of Bocillin-FL and PBP 2 by increasing amounts of penicillin G. Assays contained variable concentrations of penicillin G (0.05–1000 µM) with fixed concentrations of PBP 2 (1 µM) and Bocillin-FL (1 µM) with or without 10% DMSO. The solid line represents data for PBP 2 without DMSO and the dashed line is PBP 2 with DMSO. In all experiments, the data points represent the mean ± standard deviation of four replicate experiments at each concentration of penicillin G.

Penicillin G was used to evaluate and validate the assay for HTS. The IC_50_ values of penicillin G for PBP 2 were determined in a FP-based competition assay with and without 10% DMSO (Fig. 2) and were 1.0 µM (95% confidence interval 0.9–1.2 µM, R^2^ = 0.99) and 1.4 µM (95% confidence interval 1.2–1.5 µM, R^2^ = 0.98), respectively. The statistical parameters, including a signal-to-noise ratio >20, and Z′- and Z- factors ≥0.64, indicated the sensitivity of the FP assay and demonstrated its suitability for HTS (Table 1 and Fig. S1).

**Table 1:** Statistical parameters of the FP assay.

	Pc ± SD (mP)	Nc ± SD (mP)	S/N	Z′-factor	Dc ± SD (mP)	Z-factor
**Optimization**	172±9 (n = 8)	53±5 (n = 8)	23.8	0.65	55±5 (n = 8)	0.64
**HTS assay**	171±11 (n = 379)	46±8 (n = 368)	15.4	0.55	46.5±9.7 (n = 246)	0.51

The performance of the assay was determined during initial optimization and after the HTS assay. Pc is the mean FP signal of PBP 2-bound tracer control, Nc is the mean FP signal of free Bocillin-FL, and Dc is the FP signal in the presence of 100 µm penicillin G. S/N (signal-to-noise) and the Z factors are as defined in Material and Methods. (n = number of measurements). See also Fig. S1).

### High-throughput Assay for Inhibitors of N. gonorrhoeae PBP 2

**Figure 3:**
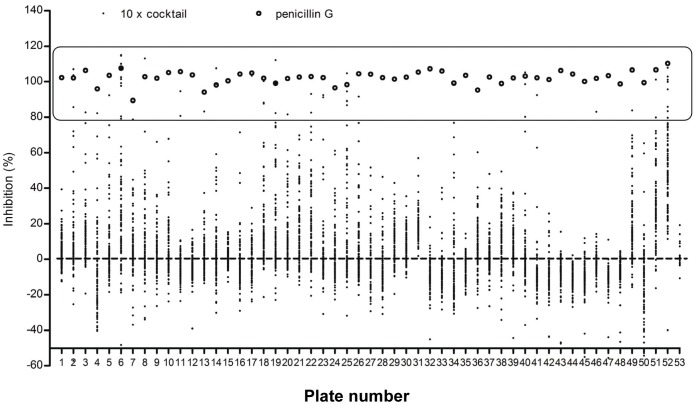
Initial HTS of 50,080 small lead compounds from DIVERSet ChemBridge Library was performed using cocktails of 10 different compounds (10X cocktails). Each plate contained duplicate samples of 10X cocktails with PBP 2 and Bocillin-FL. Data points denoted by the black dots represent the percent of inhibition for each 10X cocktail determined using the average of two sample FP measurements, and the means of free Bocillin-FL (Nc) and bound Bocillin–FL (Pc) controls recorded in quadruplicate for each corresponding plate. Data points of the displaced tracer controls (Dc - the FP of the Bocillin-FL - protein at 100 µM penicillin G), denoted by open circles, represent the percent of inhibition based on the average of four FP measurements. Fifty-two plates with 96×10X cocktails each and one plate with eight×10 X cocktails were screened. The box indicates the 58 cocktails that exhibited ≥80% inhibition of Bocillin-FL binding to PBP 2.

The FP assay was then employed in HTS of the ChemBridge DIVERset chemical library to find potential inhibitors of *N. gonorrhoeae* PBP 2. This is a diversity library of small molecules filtered for the inclusion of desirable drug-like features and exclusion of non-selective toxic groups. In one published example of HTS against zebra fish embryos using a subset of this library, the toxicity rate was 2% [34] and for the full library we have observed overall toxicity in the range of 3%, even as pooled compounds (YKP, personal observation). In the initial screening of 50,080 compounds (present as cocktails of 10 compounds), 58 cocktails exhibited ≥80% inhibition of Bocillin-FL binding to PBP 2 (Fig. 3). Each of the 580 compounds was then tested individually and 32 of these demonstrated more than 50% inhibition (Tables S1, S2). None of the “hits” were autofluorescent at the wavelengths used for the FP assay, thus paving the way for further characterization.

### Concentration-response Experiments

**Figure 4:**
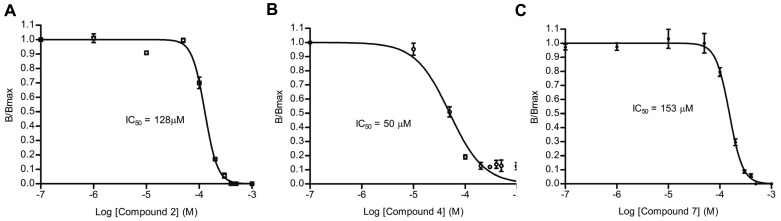
IC_50_ values of three representative hits from HTS for inhibition of acylation of PBP 2 by Bocillin-FL. IC_50_s of the inhibitors were determined by SDS-PAGE-based concentration-response assays using a 0.05–1000 µM concentration range for the inhibitors with 1 µM PBP 2 and 10 µM of Bocillin-FL. Triton X-100 (0.01%) was included to eliminate promiscuous inhibitors. A. Compound 2 (in Table 2). B. Compound 4. C. Compound 7. In all experiments, the data points represent the mean ± standard deviation for two replicate (compounds 4 and two) or four replicate (compound 7) experiments at each concentration of inhibitor.

To characterize the inhibitory activity of each of the 32 hits, concentration-response experiments were performed using the FP-based binding assay in which 0.01–200 µM of each compound was incubated with 1 µM of both PBP 2 and Bocillin-FL. Three compounds failed to show a concentration-dependent response and were excluded from further study. A cephalosporin, which had an IC_50_ of 3 µM (the lowest of all the “hits”), was also excluded since the goal was to identify non-β-lactam inhibitors; however, the successful identification of a β-lactam was validation of the effectiveness of the assay (Fig. S2 and Table S3). IC_50_ values for 24 of the remaining 28 compounds (four compounds were not available from ChemBridge) were also measured in an SDS-PAGE concentration-response assay using a 0.05–1000 µM concentration range for the inhibitor with 1 µM PBP 2 and 10 µM of Bocillin-FL to confirm their binding activities. Triton X-100 (0.01%) was included in this assay to eliminate “promiscuous” inhibitors (*e.g.* those that inhibit by non-specific aggregation [35], [36]). Six compounds failed to show concentration-dependent inhibition in the presence of Triton X-100 and were therefore excluded. The remaining 18 compounds had IC_50_ values in the range of 50–900 µM (Table 2 and Table S3). Representative examples are shown in Fig. 4 and an example of an SDS-PAGE gel used to determine the IC_50_ for compound **7** is shown in Fig. S3.

**Table 2:** IC_50_ values and antimicrobial activities of seven compounds identified by high-throughput screening against PBP 2 from *N. gonorrhoeae* strain FA19.

Compound #	IC_50_(µM; µg/ml)	MIC (µg/ml)		
		FA19	35/02	FA6140
**1**	221/135	4	16	16
**2**	128/38	4	8	16
**3**	898/208	16	32	32
**4**	50/20	16	16	16
**5**	56/20	8	16	16
**6**	49/38	4	8	8
**7**	153/54	2	8	8

### Antimicrobial Susceptibility Testing

**Figure 5:**
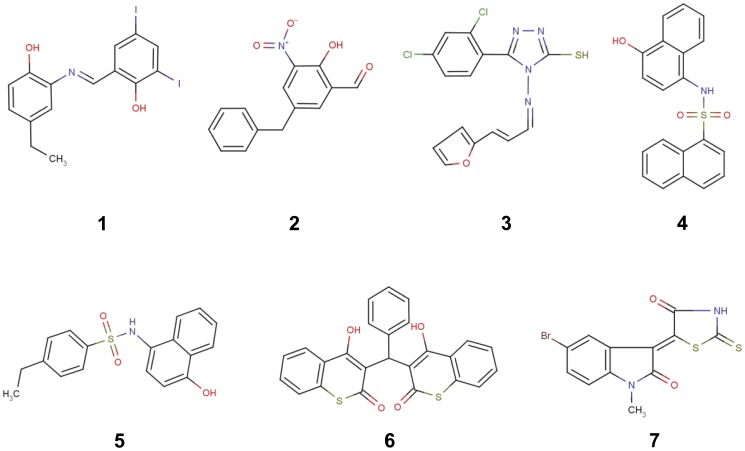
Structures of seven compounds that exhibited antimicrobial activity against *N. gonorrhoeae*. Compound numbers correspond with those in Table 2.

The 18 compounds were then tested for their ability to suppress the growth of *N. gonorrhoeae* FA19 using the disc diffusion zone method. Eleven compounds did not suppress growth and were excluded (data not shown). For the remaining 7 compounds, the minimal inhibitory concentrations (MICs) were determined against three *N. gonorrhoeae* strains: FA19, a penicillin-susceptible strain [28]; FA6140, a penicillin-resistant but cephalosporin-susceptible strain [29]; and 35/02, a strain that exhibits intermediate resistance to cephalosporin (Ceph^I^)[30]. The IC_50_ values and MICs of the seven compounds are shown in Table 2 and their respective structures are shown in Fig. 5. The MIC values ranged from 2–32 µg/ml and all except for compound **4** were higher in resistant strains compared to FA19. The best compound overall (**7**) exhibited an MIC of 2 µg/ml (5.6 µM) against FA19 and 8 µg/ml (23.4 µM) against both FA6140 and 35/02.

### Docking Simulations

**Figure 6:**
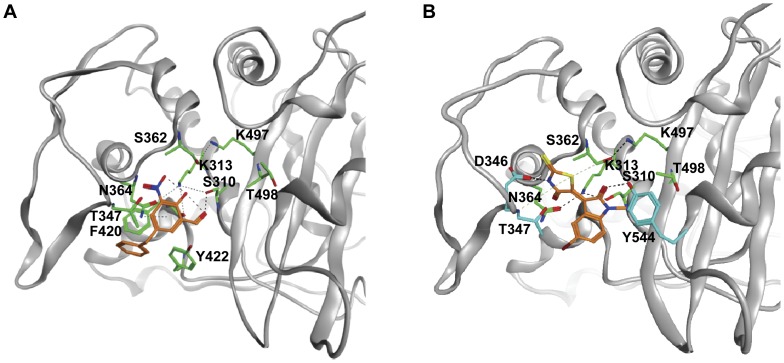
Docking of two compounds into the structure of *N. gonorrhoeae* penicillin-binding protein 2. The main chain of PBP 2 is shown as a grey ribbon, residues of the active site motifs of PBPs are shown with green bonds and additional amino acids that are predicted to form interactions with the ligand are shown in blue. **A.** compound **2**. **B.** compound **7**. Figure prepared with MOE 2011.10 (Chemical Computing Group Inc.).

In order to identify potential binding sites and evaluate the mode of interaction between the experimental compounds and PBP 2, we probed the entire surface of the protein with the inhibitors using molecular docking simulations. Five hundred random starting positions for each compound were generated and the best 250 scoring poses were refined and ranked. Top poses within the active site were energy minimized, with both protein and inhibitor bonds allowed to rotate. The presence of a refined pose within the active site of PBP 2 in the top ten poses was taken as a high probability of selective interaction (Fig. S4). Hit rates to the active site varied for each compound as follows: Compound **1** - 5/10, Compound **2** - 1/10, Compound **3** - 1/10, Compound **4** - 7/10, Compound **5** - 6/10, Compound **6** - 4/10, and Compound **7** - 3/10. These data indicate a high propensity for all the active compounds to bind to the active site. For compound **2**, the number one pose out of 250 refined poses docked within the active site (Fig. 6A). There are three predicted contacts between this compound and PBP 2: a hydrogen bond between the ligand carbonyl and the hydroxyl of Ser310 (the active site nucleophile), an interaction between the amide nitrogen of Thr347 and the nitrous acid of compound **2**, and an arene-hydrogen interaction between the conjugated phenyl ring of compound **2** and Asn364. The phenyl ring interacts with a shallow hydrophobic pocket formed by Phe420 and Tyr422 on one side, but is solvent exposed on the other. Compound **7** was found in the active site in the fifth pose out of 250 refined poses (Fig. 6B). In this model, hydrogen bonds are observed between Asp346 and the secondary amine of the thiazolidine ring, and between the hydroxyl of Tyr544 and the carbonyl of compound **7**. Additionally, there are arene-hydrogen interactions from the thiazolidine ring to both Thr347 and Ser362. The bromine atom appears to be totally solvated and does not interact with the protein directly. Overall, the docking results suggest that all of the seven compounds can bind within the active site of PBP 2, although precise details of binding can only be revealed by experimental structural studies.

## Discussion

As important targets for β-lactams and against the background of increasing clinical resistance to existing antibiotics, much effort has been directed toward developing new inhibitors of PBPs. Some approaches have sought to adapt the β-lactam moiety by, for instance, incorporating elements of the peptide substrates onto one of the R1 or R2 side chains [37], [38], [39], while others have synthesized compounds that mimic the tetrahedral intermediates of the reaction, including phosphonates and boronates [40], [41], [42], [43]. Given the wealth of structural information for several clinically important PBPs, *in silico* docking has also been employed in some systems [44]. To contribute to this effort, we have developed a high-throughput assay for PBPs that measures the fluorescence polarization (FP) of Bocillin-FL [27] and used it to screen for potential inhibitors of *N. gonorrhoeae* PBP 2 from a library of 50,080 compounds. After eliminating those that acted promiscuously, did not demonstrate a concentration-dependent inhibition, or were β-lactams, 18 compounds were examined in more detail, and 7 of these exhibited antimicrobial activity against *N. gonorrhoeae*, including Pen^R^ and Ceph^I^ strains. To the best of our knowledge, this is the first report of high-throughput screening (HTS) against a PBP.

Development of the assay was made possible by using fluorescence polarization to distinguish between the fluorescent penicillin (Bocillin-FL) remaining in solution from that bound to the PBP target, an approach first suggested by Zhao *et al.* [27]. The robustness and sensitivity of the assay is supported by the large assay window and Z factor. As a safeguard against any false positives arising from the FP-based assay, an SDS-PAGE competition assay was used to confirm inhibition of PBP 2. The identification of a cephalosporin with an IC_50_ of 3 µM against PBP2 was demonstration of the validity of the assay.

Out of the seven compounds that exhibited the lowest IC_50_ values for PBP 2 (∼ 50 µM) and good anti-gonococcal activity, two of these (compounds **4** and **5**) contain a sulfonamide group between two aromatic rings. Interestingly, a sulfonamide of similar structure was identified recently as an inhibitor of PBP2a from methicillin-resistant *Staphylococcus aureus* (MRSA) [45]. Similar to compounds **4** and **5**, compound **1** has two aromatic rings joined by a bridging group reminiscent of the sulfonamide inhibitors and showed reasonable antimicrobial activity against FA19, but less so against 6140 and 35/02. Compound **2** also possesses two aromatic rings, one of which contains a potentially reactive hydroxyl nitrobenzaldehyde group. This compound demonstrated moderate antibacterial activity against FA19, with higher MIC values against FA610 and 35/02. It was the most successful compound during the docking simulations and in this model there is an interaction with the serine nucleophile of PBP 2 (Ser310). Compound **6** exhibited the lowest IC_50_ value (49 µM) with good MICs against FA19 (4 µg/ml) and the two antibiotic-resistant strains (both were 8 µg/ml). Compound **7** showed rather moderate enzyme inhibition (IC_50_ = 153 µM) but exhibited the highest antimicrobial activity in all *N. gonorrhoeae* strains. Interestingly, this compound contains a thiazolidine ring, a feature of penicillin antibiotics and arylalkylidene iminothiazolidin-4-ones, which inhibit some PBPs *in vitro* and shows antimicrobial activity [46]. Compound **7** was also successful in the docking simulations and there are predicted interactions with Ser310. The resemblance of compound **7** to a β-lactam-like compound led us to determine whether there is a covalent interaction with PBP 2, but no increase in mass of the protein was observed by mass spectrometry (data not shown). Finally, compound **3** is probably the least promising candidate at this stage since it has a relatively high IC_50_ against PBP 2 and its MIC values against the *N. gonorrhoeae* strains are correspondingly weak.

Overall, there was relatively weak correlation between IC_50_ and MIC values for the seven compounds, but this is expected for initial hits from HTS because there are a variety of factors that determine the MIC. Since *N. gonorrhoeae* expresses two essential PBPs, PBP 1 could be the lethal target for a given compound in addition to, or independent of, PBP 2. The outer membrane of Gram-negative bacteria is a barrier to hydrophobic compounds [47] and the lipooligosaccharide structure of *N. gonorrhoeae* also impacts permeability. In addition, entry of hydrophilic compounds into the periplasm is influenced by porins and the action of efflux systems can remove compounds from the periplasm. Both of the antibiotic resistant strains (FA6140 and 35/02) contain a mutation in the promoter of *mtrCDE* that increases expression of the MtrC-MtrD-MtrE efflux pump. In addition, both strains harbor an altered *porB_1B_* allele encoding the major outer membrane porin, which decreases influx of antibiotics into the periplasmic space [17], [30], [48]. Hence, the ability of the different compounds to permeate porins or to be substrates of the efflux pump can have a profound impact on antibiotic efficacy [19]. It is also possible that some of the compounds with low MICs are cytotoxic to *N. gonorrhoeae*. Further investigation of the compounds is therefore necessary to establish their *in vivo* mechanism of antimicrobial activity.

In conclusion, an FP-based HTS has generated several inhibitors of PBP 2 and several show promising antimicrobial activity against Pen^R^ and Ceph^I^ strains of *N. gonorrhoeae*. Such compounds require further study to confirm their mechanism of action, followed by chemical optimization to improve their efficacy. The development of this assay paves the way to screen using larger compound libraries and against variants of PBP 2 that contribute to third-generation cephalosporin resistance.

## Supporting Information

Figure S1 Millipolarization values (mP) of positive control (Pc), negative control (Nc) and displaced tracer control (Dc) for all measurements recorded during the HTS, including the initial optimization. The data points represent mP measurements (n = sample number).
(TIF)

Figure S2 Four of the 32 compounds that were excluded from further study. **A.** Compounds 30, 31, 32 failed to show a concentration-dependent response in FP-based experiments. IC_50_s of the compounds were determined by FP-based concentration-response assays using a 0.01–200 µM concentration range for the compounds with 1 µM PBP 2 and 1 µM of Bocillin-FL. In all experiments, the data points represent the mean ± standard deviation over four replicate experiments. **B.** Compound 29 had an IC_50_ of 3 µM (the lowest of all the “hits”), but is a cephalosporin.
(TIF)

Figure S3 SDS-PAGE-based analysis of the inhibitory activity of compound **7** against PBP 2 (IC_50_ = 153 µM). PBP 2 (1 µM) in 50 mM sodium phosphate, 0.01% Triton X-100, pH 8 was incubated with 0.05–1000 µM of compound **7** for 1 h, followed by 15 min incubation with 10 µM Bocillin-FL. The reaction was stopped by mixing with 5 X SDS-loading buffer, followed by boiling for 2 min. 10% SDS-PAGE gels was then used to separate bound PBP 2 from free ligand. At least two independent reactions were performed in duplicate at each concentration of the inhibitor. Gels were visualized by UV (top panel) to measure IC_50_ and the same gels were stained with Coomassie Brilliant Blue R-250 to verify equal loading of protein (lower panel).
(TIF)

Figure S4 Docking of compounds as surface probes with PBP 2. Depicted are the top 10 poses for each compound (numbered 1–7) from 250 refined poses, as described in the Material & Methods. PBP 2 is displayed as a grey surface and is in the same orientation as Fig. 6. The active site region is colored green and the compounds are displayed in bond format and colored orange.
(TIF)

Table S1 The layout of the 384-well plates used for the high-throughput screening of the 50,080 compound Chembridge DIVERSet library. Compounds were present as cocktails of 10 compounds each (10X cocktails). In total, fifty-two plates with 96 cocktails and one plate with 8 cocktails were screened. The wells are numbered according to the scheme below, where each well contains one cocktail and each cocktail is present twice for two independent measurements. Rows J-P in each plate were not used. Dc = displaced tracer control, Bk = blank, Nc = negative control and Pc = positive control.
(DOCX)

Table S2 Analysis of the 58 cocktails showing ≥80% inhibition of Bocillin-FL binding to PBP 2.
(DOCX)

Table S3 Analysis of 32 individual compounds that exhibited ≥50% inhibition of Bocillin-FL binding to PBP 2.
(DOCX)
