# Supplementary figures and images for: High-Throughput Screening for Novel Inhibitors of Neisseria gonorrhoeae Penicillin-Binding Protein 2
Source: PLoS One. 2012 Sep 25;7(9):e44918. doi: 10.1371/journal.pone.0044918 (PMC3458020; doi:10.1371/journal.pone.0044918)

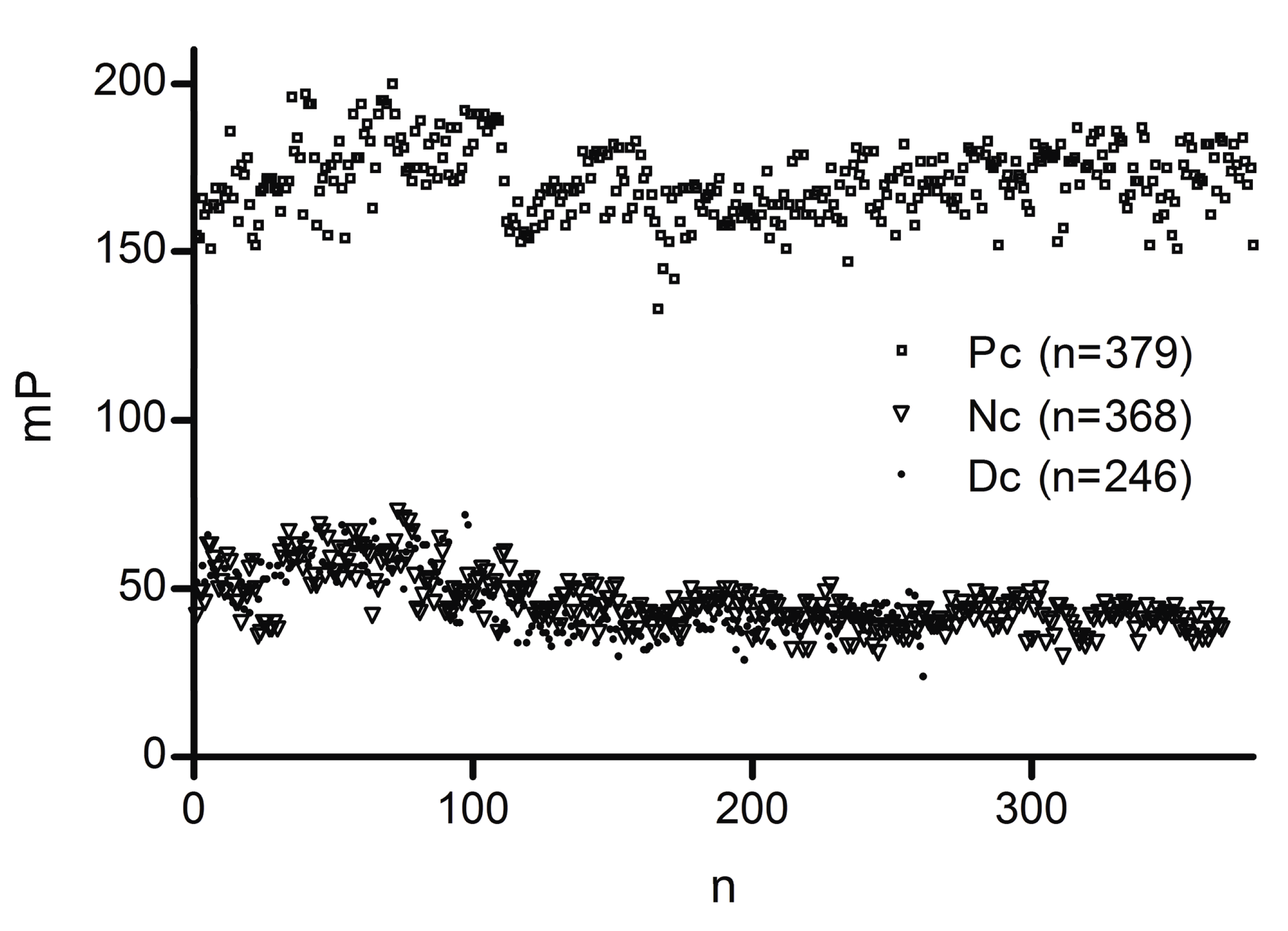

Supplement: Figure S1 — Millipolarization values (mP) of positive control (Pc), negative control (Nc) and displaced tracer control (Dc) for all measurements recorded during the HTS, including the initial optimization. The data points represent mP measurements (n = sample number). (TIF) [file pone.0044918.s001.tif]

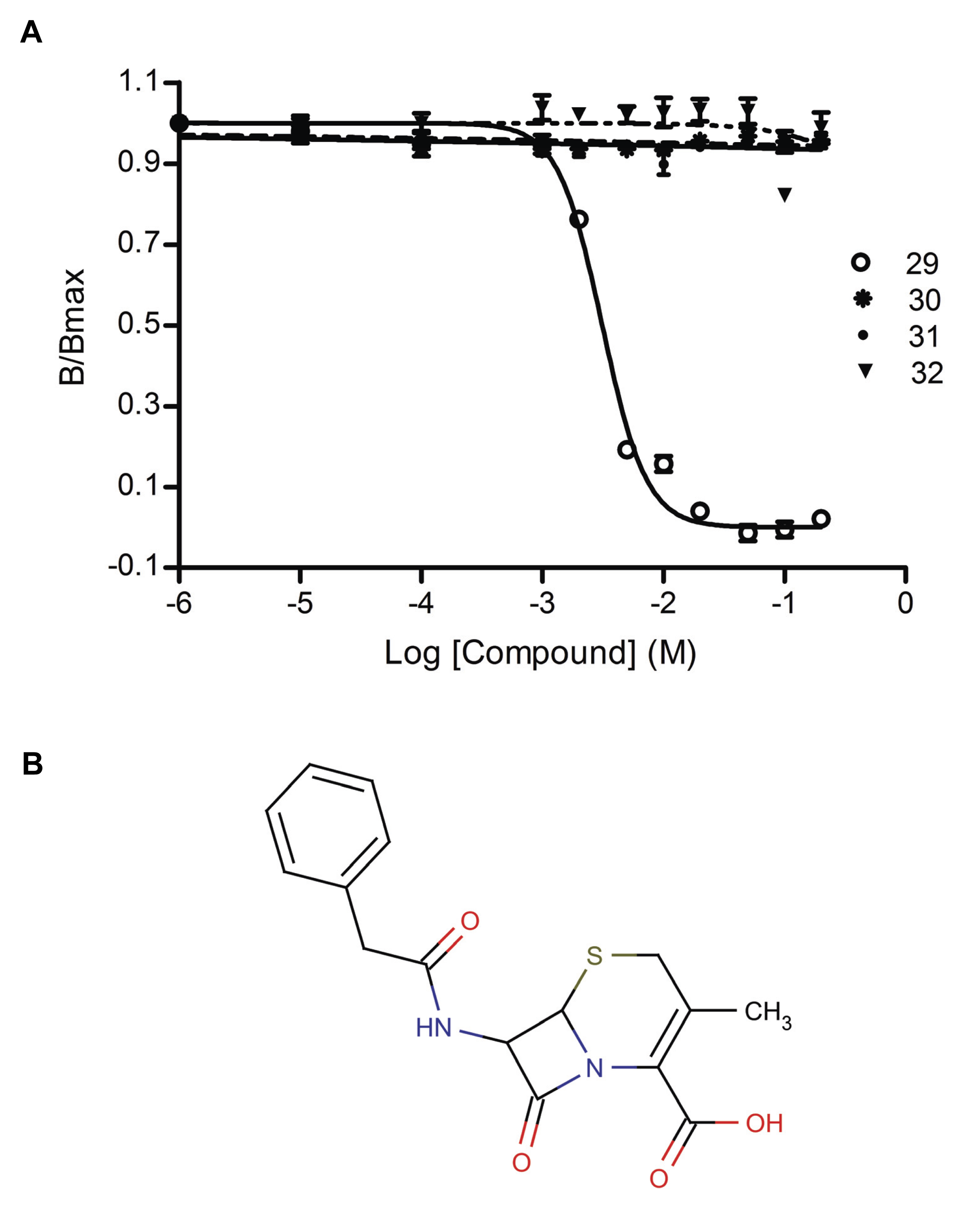

Supplement: Figure S2 — Four of the 32 compounds that were excluded from further study. A. Compounds 30, 31, 32 failed to show a concentration-dependent response in FP-based experiments. IC50s of the compounds were determined by FP-based concentration-response assays using a 0.01–200 µM concentration range for the compounds with 1 µM PBP 2 and 1 µM of Bocillin-FL. In all experiments, the data points represent the mean ± standard deviation over four replicate experiments. B. Compound 29 had an IC50 of 3 µM (the lowest of all the “hits”), but is a cephalosporin. (TIF) [file pone.0044918.s002.tif]

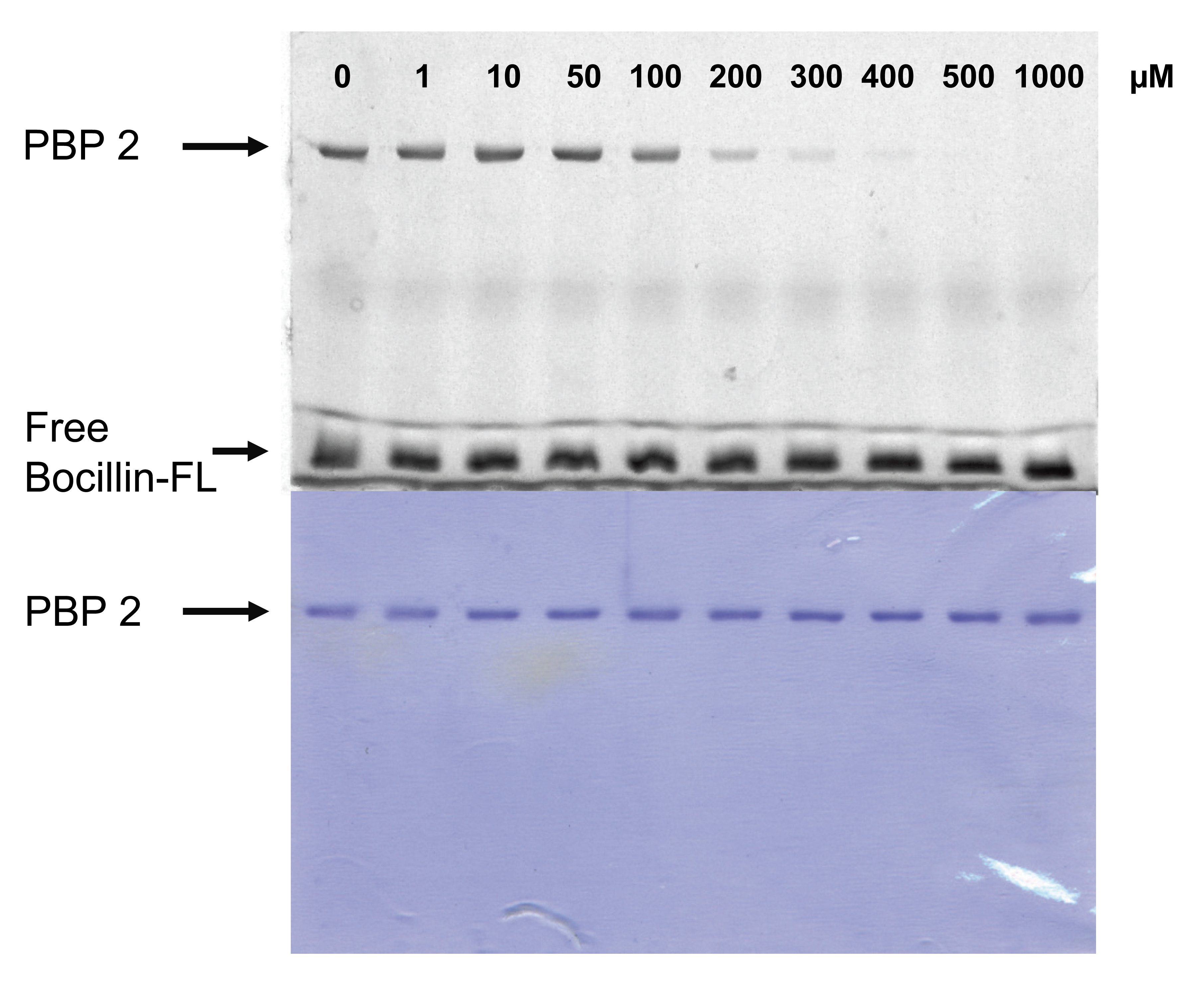

Supplement: Figure S3 — SDS-PAGE-based analysis of the inhibitory activity of compound 7 against PBP 2 (IC50 = 153 µM). PBP 2 (1 µM) in 50 mM sodium phosphate, 0.01% Triton X-100, pH 8 was incubated with 0.05–1000 µM of compound 7 for 1 h, followed by 15 min incubation with 10 µM Bocillin-FL. The reaction was stopped by mixing with 5 X SDS-loading buffer, followed by boiling for 2 min. 10% SDS-PAGE gels was then used to separate bound PBP 2 from free ligand. At least two independent reactions were performed in duplicate at each concentration of the inhibitor. Gels were visualized by UV (top panel) to measure IC50 and the same gels were stained with Coomassie Brilliant Blue R-250 to verify equal loading of protein (lower panel). (TIF) [file pone.0044918.s003.tif]

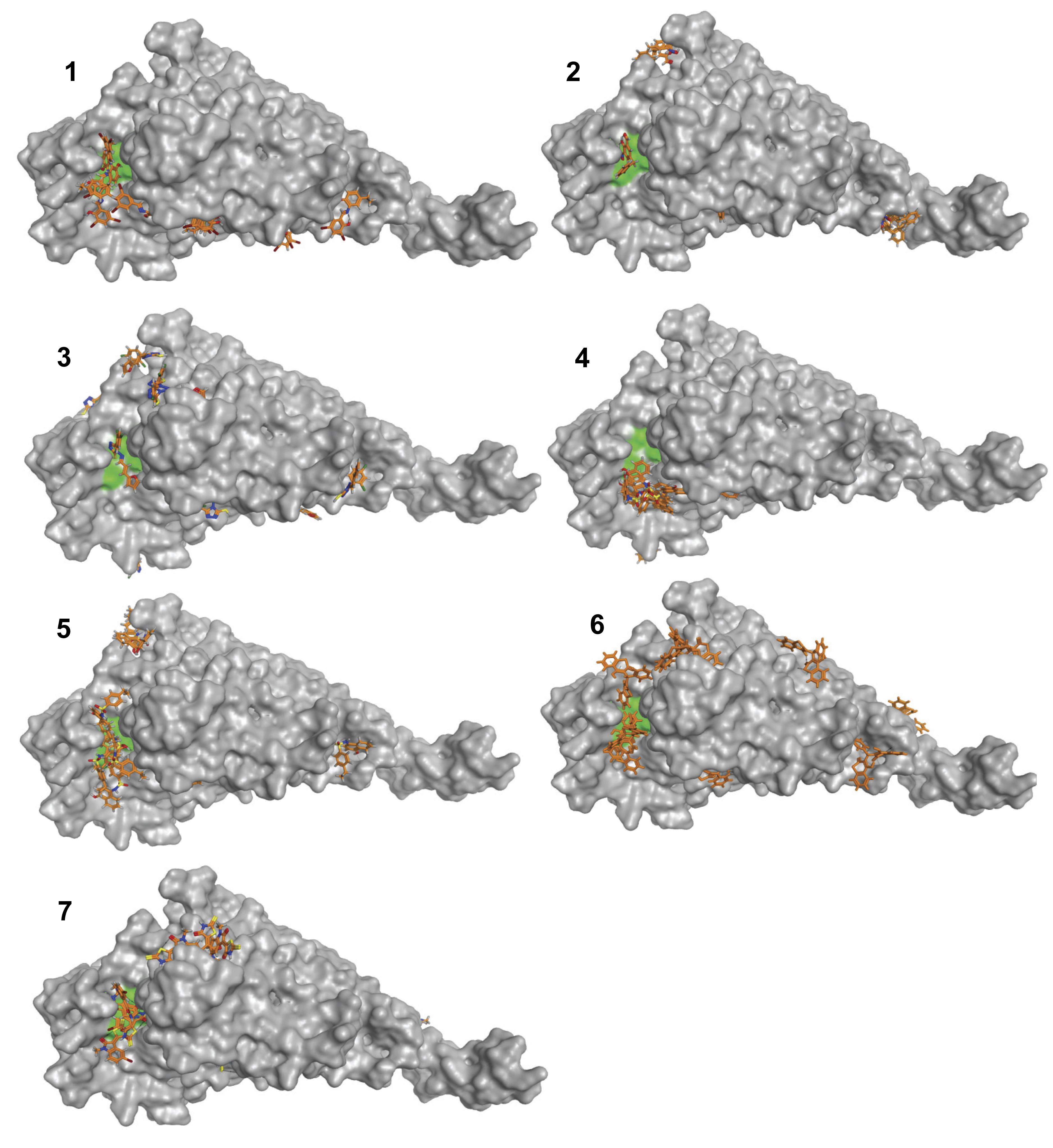

Supplement: Figure S4 — Docking of compounds as surface probes with PBP 2. Depicted are the top 10 poses for each compound (numbered 1–7) from 250 refined poses, as described in the Material & Methods. PBP 2 is displayed as a grey surface and is in the same orientation as Fig. 6. The active site region is colored green and the compounds are displayed in bond format and colored orange. (TIF) [file pone.0044918.s004.tif]
