# Supplementary material for: High-Throughput Screening for Novel Inhibitors of Neisseria gonorrhoeae Penicillin-Binding Protein 2
Source: PLoS One. 2012 Sep 25;7(9):e44918. doi: 10.1371/journal.pone.0044918 (PMC3458020; doi:10.1371/journal.pone.0044918)
Supplement: Table S1 — The layout of the 384-well plates used for the high-throughput screening of the 50,080 compound Chembridge DIVERSet library. Compounds were present as cocktails of 10 compounds each (10X cocktails). In total, fifty-two plates with 96 cocktails and one plate with 8 cocktails were screened. The wells are numbered according to the scheme below, where each well contains one cocktail and each cocktail is present twice for two independent measurements. Rows J-P in each plate were not used. Dc = displaced tracer control, Bk = blank, Nc = negative control and Pc = positive control. (DOCX) [file pone.0044918.s005.docx]

|  | **1** | **2** | **3** | **4** | **5** | **6** | **7** | **8** | **9** | **10** | **11** | **12** | **13** | **14** | **15** | **16** | **17** | **18** | **19** | **20** | **21** | **22** | **23** | **24** |
| --- | --- | --- | --- | --- | --- | --- | --- | --- | --- | --- | --- | --- | --- | --- | --- | --- | --- | --- | --- | --- | --- | --- | --- | --- |
| **A** | A1 | A1 | A2 | A2 | A3 | A3 | A4 | A4 | A5 | A5 | A6 | A6 | A7 | A7 | A8 | A8 | A9 | A9 | A10 | A10 | A11 | A11 | A12 | A12 |
| **B** | B1 | B1 | B2 | B2 | B3 | B3 | B4 | B4 | B5 | B5 | B6 | B6 | B7 | B7 | B8 | B8 | B9 | B9 | B10 | B10 | B11 | B11 | B12 | B12 |
| **C** | C1 | C1 | C2 | C2 | C3 | C3 | C4 | C4 | C5 | C5 | C6 | C6 | C7 | C7 | C8 | C8 | C9 | C9 | C10 | C10 | C11 | C11 | C12 | C12 |
| **D** | D1 | D1 | D2 | D2 | D3 | D3 | D4 | D4 | D5 | D5 | D6 | D6 | D7 | D7 | D8 | D8 | D9 | D9 | D10 | D10 | D11 | D11 | D12 | D12 |
| **E** | E1 | E1 | E2 | E2 | E3 | E3 | E4 | E4 | E5 | E5 | E6 | E6 | E7 | E7 | E8 | E8 | E9 | E9 | E10 | E10 | E11 | E11 | E12 | E12 |
| **F** | F1 | F1 | F2 | F2 | F3 | F3 | F4 | F4 | F5 | F5 | F6 | F6 | F7 | F7 | F8 | F8 | F9 | F9 | F10 | F10 | F11 | F11 | F12 | F12 |
| **G** | G1 | G1 | G2 | G2 | G3 | G3 | G4 | G4 | G5 | G5 | G6 | G6 | G7 | G7 | G8 | G8 | G9 | G9 | G10 | G10 | G11 | G11 | G12 | G12 |
| **H** | H1 | H1 | H2 | H2 | H3 | H3 | H4 | H4 | H5 | H5 | H6 | H6 | H7 | H7 | H8 | H8 | H9 | H9 | H10 | H10 | H11 | H11 | H12 | H12 |
| **I** | Dc | Dc | Dc | Dc | Bk | Bk | Bk | Bk | Nc | Nc | Nc | Nc | Pc | Pc | Pc | Pc |  |  |  |  |  |  |  |  |
| **J** |  |  |  |  |  |  |  |  |  |  |  |  |  |  |  |  |  |  |  |  |  |  |  |  |
| **K** |  |  |  |  |  |  |  |  |  |  |  |  |  |  |  |  |  |  |  |  |  |  |  |  |
| **L** |  |  |  |  |  |  |  |  |  |  |  |  |  |  |  |  |  |  |  |  |  |  |  |  |
| **M** |  |  |  |  |  |  |  |  |  |  |  |  |  |  |  |  |  |  |  |  |  |  |  |  |
| **N** |  |  |  |  |  |  |  |  |  |  |  |  |  |  |  |  |  |  |  |  |  |  |  |  |
| **O** |  |  |  |  |  |  |  |  |  |  |  |  |  |  |  |  |  |  |  |  |  |  |  |  |
| **P** |  |  |  |  |  |  |  |  |  |  |  |  |  |  |  |  |  |  |  |  |  |  |  |  |
