# Supplementary material for: High-Throughput Screening for Novel Inhibitors of Neisseria gonorrhoeae Penicillin-Binding Protein 2
Source: PLoS One. 2012 Sep 25;7(9):e44918. doi: 10.1371/journal.pone.0044918 (PMC3458020; doi:10.1371/journal.pone.0044918)
Supplement: Table S2 — Analysis of the 58 cocktails showing ≥80% inhibition of Bocillin-FL binding to PBP 2. (DOCX) [file pone.0044918.s006.docx]

|  | **Controls/plate** | |  | | |  |
| --- | --- | --- | --- | --- | --- | --- |
| **Plate number** | **Nc (mP)* (mean ± SD)** | **Pc (mP)* (mean ± SD)** | **Well position** | **Cocktails (mP)** (mean ± SD)** | **Inhibition (%)** | **Individual hits with ≥50% inhibition** |
|  |  |  |  |  |  |  |
| 2 | 66.1 ± 7.2 | 186.2 ± 2.6 | A1 | 61.6 ± 13.3 | 104 | Y |
| 2 | “ | “ | A10 | 83.2 ± 4.9 | 86 | Y |
| 3 | 62.5 ± 7.4 | 185.7 ± 4.2 | C2 | 84.1 ± 7.5 | 83 | - |
| 4 | 73.3 ± 12 | 201.1 ± 18 | B11 | 96.0 ± 2.4 | 82 | Y |
| 5 | 44.8 ± 1.4 | 172.5 ± 1.0 | B6 | 54.6 ± 3.7 | 92 | - |
| 6 | 67.0 ± 11.5 | 176.0 ± 10 | A4 | 50.7 ± 0.5 | 115 | - |
| 6 | “ | “ | B4 | 59.3 ± 0.3 | 107 | - |
| 6 | “ | “ | B10 | 65.4 ± 0.6 | 101 | Y |
| 6 | “ | “ | C1 | 89.7 ± 14.8 | 80 | - |
| 6 | “ | “ | C4 | 59.6 ± 4.0 | 107 | - |
| 6 | “ | “ | C10 | 64.2 ± 9.2 | 103 | Y |
| 6 | “ | “ | D3 | 51.1 ± 9.0 | 115 | - |
| 6 | “ | “ | D10 | 56.1 ± 0.1 | 110 | Y |
| 6 | “ | “ | F10 | 69.5 ± 6.8 | 98 | Y |
| 6 | “ | “ | G10 | 59.7 ± 2.4 | 107 | - |
| 6 | “ | “ | G12 | 67.9 ± 0.6 | 99 | - |
| 6 | “ | “ | H4 | 88.3 ± 5.3 | 81 | - |
| 6 | “ | “ | H5 | 56.0 ± 2.4 | 110 | - |
| 7 | 50.6 ± 6.6 | 177.7 ± 0.6 | A9 | 78.0 ± 12.1 | 80 | Y |
| 8 | 48.6 ± 4.6 | 166.0 ± 4.4 | B5 | 33.2 ± 11.1 | 113 | Y |
| 11 | 49.2 ± 3.6 | 157.6 ± 0.7 | H8 | 52.8 ± 0.8 | 95 | Y |
| 11a | 46.8 ± 0.9 | 164.4 ± 3.8 | C1 | 75.0 ± 6.7 | 81 | - |
| 13 | 59.0 ± 4.3 | 183.5 ± 5.7 | C12 | 79.9 ± 11.7 | 83 | Y |
| 14 | 60.5 ± 7.2 | 187.1 ± 8.4 | B3 | 62.6 ± 10.6 | 99 | Y |
| 14 | “ | “ | F7 | 60.3 ± 3.0 | 108 | Y |
| 17 | 43.4 ± 3.5 | 172.6 ± 10.3 | D8 | 39.0 ± 1.4 | 103 | Y |
| 18 | 39.2 ± 1.9 | 181.8 ± 0.2 | B11 | 55.9 ± 2.1 | 88 | Y |
| 18 | “ | “ | C8 | 38.7 ± 6.0 | 100 | Y |
| 18 | “ | “ | D10 | 56.7 ± 3.7 | 88 | Y |
| 19 | 36.7 ± 3.3 | 179.1 ± 6.1 | B2 | 36.4 ± 4.9 | 100 | Y |
| 19 | “ | “ | B11 | 61.9 ± 2.3 | 82 | Y |
| 19 | “ | “ | F8 | 37.8 ± 7.1 | 99 | Y |
| 19 | “ | “ | F12 | 19.3 ± 1.5 | 112 | Y |
| 20 | 41.6 ± 2.2 | 172.9 ± 7.4 | F12 | 65.9 ± 8.0 | 82 | - |
| 22 | 40.1 ± 2.3 | 169.3 ± 13.8 | B2 | 62.6 ± 5.7 | 83 | - |
| 22 | “ | “ | B6 | 49.0 ± 3.9 | 93 | - |
| 23 | 42.4 ± 0.7 | 174.0 ± 0.2 | A10 | 52.3 ± 3.5 | 92 | Y |
| 23 | “ | “ | B12 | 54.1 ± 2.9 | 91 | - |
| 25 | 38.6 ± 0.9 | 174.0 ± 4.6 | C2 | 46.0 ± 0.6 | 95 | Y |
| 25 | “ | “ | E4 | 53.3 ± 2.2 | 89 | - |
| 25 | “ | “ | F3 | 56.4 ± 21.1 | 87 | - |
| 25 | “ | “ | G2 | 32.2 ± 1.8 | 105 | Y |
| 26 | 46.2 ± 1.3 | 172.0 ± 2.0 | B6 | 56.8 ± 3.6 | 92 | - |
| 34 | 46.0 ± 4.2 | 158.0 ± 7.3 | F9 | 47.7 ± 4.0 | 98 | Y |
| 34 | “ | “ | G6 | 69.0 ± 2.4 | 80 | - |
| 40 | 43.3 ± 3.1 | 169.0 ± 1.8 | C9 | 68.3 ± 1.1 | 80 | Y |
| 41 | 40.7 ± 1.8 | 165.5 ± 2.8 | B3 | 50.2 ± 1.0 | 92 | Y |
| 46 | 37.6 ± 0.4 | 171.8 ± 0.2 | E11 | 60.3 ± 9.3 | 83 | Y |
| 49 | 67.0 ± 10.3 | 177.0 ± 4.9 | F12 | 84.8 ± 0.7 | 84 | - |
| 51 | 66.0 ± 12.5 | 191.0 ± 8.6 | C11 | 91.2 ± 33.8 | 80 | - |
| 51 | “ | “ | H12 | 64.5 ± 2.8 | 101 | - |
| 52 | 78.0 ± 22.0 | 219.0 ± 48 | B8 | 78.2 ± 21.1 | 100 | Y |
| 52 | “ | “ | C5 | 76.5 ± 8.7 | 101 | Y |
| 52 | “ | “ | E2 | 106.7 ± 4.2 | 80 | - |
| 52 | “ | “ | E6 | 84.0 ± 49.1 | 96 | Y |
| 52 | “ | “ | E8 | 74.0 ± 5.3 | 103 | Y |
| 52 | “ | “ | F11 | 67.0 ± 5.1 | 108 | - |
| 52 | “ | “ | H1 | 101.4 ± 17.3 | 83 | - |

* Values determined based on the data from four replicate FP readings of controls

** Values determined based on the data from two replicate FP readings
