# Supplementary material for: High-Throughput Screening for Novel Inhibitors of Neisseria gonorrhoeae Penicillin-Binding Protein 2
Source: PLoS One. 2012 Sep 25;7(9):e44918. doi: 10.1371/journal.pone.0044918 (PMC3458020; doi:10.1371/journal.pone.0044918)
Supplement: Table S3 — Analysis of 32 individual compounds that exhibited ≥50% inhibition of Bocillin-FL binding to PBP 2. (DOCX) [file pone.0044918.s007.docx]

**Table S3:** Analysis of 32 individual compounds that exhibited ≥50% inhibition of Bocillin-FL binding to PBP 2

| **Compound number** | **Plate number /well position** | **ChemBridge ID** | **M.W.** | **Nc (mP)* (mean ± SD)** | **Pc (mP)* (mean ± SD)** | **Compound (mP)* (mean ± SD)** | **Inhibition (%)*** | **IC_50_ measured by FP (µM)*** | **IC_50_ measured by SDS-PAGE (µM)**** |
| --- | --- | --- | --- | --- | --- | --- | --- | --- | --- |
| **1** | 2/A1 | 5477031 | 493 | 42.0±3.3 | 172.0±11.8 | 31.4±0.1 | 108.2 | 113.7 | 221.4 |
| **2** | 2/A10 | 5530695 | 257 | 42.0±3.3 | 172.0±11.8 | 60.4±20.3 | 85.8 | 330.4 | 127.9 |
| **3** | 4/B11 | 6624338 | 365 | 42.0±3.3 | 172.0±11.8 | 65.7±12.4 | 81.8 | 128.8 | 898 |
| **4** | 11/H8 | 7647715 | 349 | 43.0±3.6 | 181.0±4.4 | 35.6±2.1 | 105.4 | 51.4 | 50.22 |
| **5** | 14/B3 | 5704102 | 327 | 40.0±3.8 | 179.0±15.6 | 43.3±0.8 | 97.6 | 52.1 | 56.26 |
| **6** | 52/C5 | 5151615 | 445 | 40.0±3.8 | 179.0±15.6 | 54.6±3.2 | 89.5 | 50.9 | 49.34 |
| **7** | 18/C8 | 5201291 | 355 | 41.0±2.0 | 165.0±17.7 | 45.2±20.2 | 96.6 | 11.3 | 152.8 |
| **8** | 6/C10 | 5730506 | 423 | 40.0±4.5 | 171.0±10.8 | 35.4±3.7 | 95.9 | 22.2 | 230 |
| **9** | 6/D10 | 5729373 | 365 | 40.0±4.5 | 171.0±10.8 | 27.8±11.7 | 109.3 | 14.7 | 442 |
| **10** | 6/F10 | 5737652 | 393 | 46.0±1.3 | 173.0±6.6 | 84.1±42.0 | 62.7 | 39.9 | 109 |
| **11** | 17/D8 | 7630081 | 398 | 41.0±2.0 | 165.0±17.7 | 32.3±5.1 | 107.0 | 63.7 | 248 |
| **12** | 18/B11 | 5214931 | 354 | 41.0±2.0 | 165.0±17.7 | 54.0±8.0 | 89.5 | 66.1 | 182 |
| **13** | 19/B2 | 5218577 | 351 | 42.0±1.8 | 168.0±12.7 | 33.5±0.5 | 106.7 | 53.7 | 59 |
| **14** | 19/B11 | 5247550 | 398 | 42.0±1.8 | 168.0±12.7 | 68.6±10.8 | 79.0 | 247.0 | 224 |
| **15** | 19/F8 | 5238280 | 363 | 42.0±1.8 | 168.0±12.7 | 36.8±3.5 | 104.1 | 67.8 | 534 |
| **16** | 25/C2 | 5376753 | 367 | 41.0±3.5 | 168.0±1.9 | 37.1±11.1 | 103.0 | 41.5 | 144 |
| **17** | 25/G2 | 5376658 | 339 | 41.0±3.5 | 168.0±1.9 | 31.6±1.0 | 107.4 | 32.5 | 598 |
| **18** | 52/E8 | 5175181 | 317 | 37±2.4 | 173.3±0.8 | 66±19.1 | 79.0 | 91.8 | 273 |
| **19** | 6/B10 | 5729311 | 348 | 40.0±4.5 | 171.0±10.8 | 71.6±11.4 | 75.9 | 169.2 | NR |
| **20** | 8/B5 | 5667569 | 287 | 43.0±3.6 | 181.0±4.4 | 34.8±0.5 | 106.0 | 13.8 | NR |
| **21** | 19/F12 | 5255358 | 412 | 42.0±1.8 | 168.0±12.7 | 53.0±8.5 | 91.4 | 573.4 | NR |
| **22** | 46/E11 | 9039164 | 352 | 37.5±1.6 | 162.4±1.5 | 38.7±3.3 | 99.1 | 34.6 | NR |
| **23** | 52/B8 | 5180253 | 230 | 37±2.4 | 173.3±0.8 | 73.6±7.1 | 73.0 | 67.4 | NR |
| **24** | 52/E6 | 5155500 | 361 | 42.0±3.3 | 172.0±11.8 | 58.9±5.5 | 87.0 | 348.4 | NR |
| **25** | 7/A9 | 5841052 | 400 | 43.0±3.6 | 181.0±4.4 | 51.8±4.5 | 93.6 | 52.3 | UN |
| **26** | 13/C12 | 5535385 | 329 | 40.0±3.8 | 179.0±15.6 | 38.1±4.9 | 101.4 | 37.0 | UN |
| **27** | 14/F7 | 5947475 | 309 | 41.0±2.0 | 165.0±17.7 | 46.3±0.5 | 95.7 | 71.2 | UN |
| **28** | 34/F9 | 7708424 | 330 | 40.0±3.8 | 179.0±15.6 | 36.6±0.4 | 102.4 | 10.8 | UN |
| **29** | 41/B3 | 7817085 | 332 | 41.0±3.5 | 168.0±1.9 | 51.8±3.7 | 90.7 | 3.1 | CEPH |
| **30** | 18/D10 | - |  | 41.0±2.0 | 165.0±17.7 | 77.0±30.0 | 71.0 | No response |  |
| **31** | 23/A10 | - |  | 42.0±1.8 | 168.0±12.7 | 86.5±2.8 | 64.9 | No response |  |
| **32** | 40/C9 | - |  | 41.0±3.5 | 168.0±1.9 | 51.9±9.6 | 91.4 | No response |  |

* Values determined with data from four replicate experiments

** SDS-PAGE-based concentration-response experiments were performed in the presence of 0.01% Triton X-100. IC_50_ values were determined from two independent experiments with two gels for each.

NR=no concentration response, UN=compound unavailable, CEPH=the compound identified was a cephalosporin
